# DrosOMA: the *Drosophila* Orthologous Matrix browser

**DOI:** 10.12688/f1000research.135250.1

**Published:** 2023-08-07

**Authors:** Antonin Thiébaut, Adrian M. Altenhoff, Giulia Campli, Natasha Glover, Christophe Dessimoz, Robert M. Waterhouse

**Affiliations:** 1 Department of Ecology and Evolution, SIB Swiss Institute of Bioinformatics, University of Lausanne, Lausanne, Switzerland; 2 Department of Computer Science, SIB Swiss Institute of Bioinformatics, ETH Zurich, Zurich, Switzerland; 3 Department of Computational Biology, SIB Swiss Institute of Bioinformatics, University of Lausanne, Lausanne, Switzerland

**Keywords:** Drosophila, orthology, orthologues, comparative genomics, database, orthologous groups, gene families, synteny

## Abstract

**Background:** Comparative genomic analyses to delineate gene evolutionary histories inform the understanding of organismal biology by characterising gene and gene family origins, trajectories, and dynamics, as well as enabling the tracing of speciation, duplication, and loss events, and facilitating the transfer of gene functional information across species. Genomic data are available for an increasing number of species from the genus Drosophila, however, a dedicated resource exploiting these data to provide the research community with browsable results from genus-wide orthology delineation has been lacking.

**Methods:** Using the OMA Orthologous Matrix orthology inference approach and browser deployment framework, we catalogued orthologues across a selected set of Drosophila species with high-quality annotated genomes. We developed and deployed a dedicated instance of the OMA browser to facilitate intuitive exploration, visualisation, and downloading of the genus-wide orthology delineation results.

**Results:** DrosOMA - the Drosophila Orthologous Matrix browser, accessible from https://drosoma.dcsr.unil.ch/ - presents the results of orthology delineation for 36 drosophilids from across the genus and four outgroup dipterans. It enables querying and browsing of the orthology data through a feature-rich web interface, with gene-view, orthologous group-view, and genome-view pages, including comprehensive gene name and identifier cross-references together with available functional annotations and protein domain architectures, as well as tools to visualise local and global synteny conservation.

**Conclusions:** The DrosOMA browser demonstrates the deployability of the OMA browser framework for building user-friendly orthology databases with dense sampling of a selected taxonomic group. It provides the Drosophila research community with a tailored resource of browsable results from genus-wide orthology delineation.

## Introduction

The fruit fly, *Drosophila melanogaster*, is one of the most comprehensively studied model organisms, supported by decades of research, with advanced genetic tools and genomic resources, and a wealth of accumulated knowledge ( Adams *et al*. 2000; Markow 2015). It is therefore a key source of gene functional information that can be tentatively propagated to other species through an evolutionarily-informed framework. Reciprocally, cross-species genomic comparisons help to delineate gene evolutionary histories and thereby further inform *D. melanogaster* biology by characterising gene and gene family origins, trajectories, and dynamics. This is evident from early cross-phyla perspectives ( Rubin *et al*. 2000; Venter *et al*. 2001) and over shorter evolutionary timescales such as across the *Drosophila* genus ( Drosophila 12 Genomes Consortium 2007; Hahn *et al*. 2007; Heger and Ponting 2007). Continued sequencing efforts e.g. ( Kim *et al*. 2021; Suvorov *et al*. 2022) mean that genome assemblies are now available for some 150 *Drosophila* species, providing unprecedented resolution for employing comparative approaches to study gene and genome evolution across the genus.

Cross-species comparisons to characterise gene evolutionary histories provide a foundation from which to trace speciation, duplication, and loss events leading to the gene repertoires encoded in each species’ genome ( Koonin 2005). Arising respectively through speciation and duplication events, orthologues and paralogues together form orthologous groups comprising all genes descended from a single gene in the last common ancestor of the set of species under consideration ( Nevers *et al*. 2020). Numerous methods, broadly categorised as tree-based or graph-based approaches, have been developed to delineate orthologous groups ( Altenhoff and Dessimoz 2012), with ongoing efforts to improve quality and scalability of orthology resources ( Linard *et al*. 2021; Nevers *et al*. 2022). Such resources provide the basis for building evolutionarily-informed hypotheses on gene function, or the so-called transfer of functional annotations. This relies on the baseline assumption of functional equivalency amongst genes that share a common ancestor, which although not without its caveats ( Robinson-Rechavi 2020), remains the primary means of large-scale functional annotations.

As the primary database for researchers using *D. melanogaster* as a model organism, FlyBase provides access to a wide range of information including genetic, genomic, molecular, and reagent resources ( Larkin *et al*. 2021; Gramates *et al*. 2022). For cross-species gene repertoire comparisons, FlyBase employs the *Drosophila* RNAi Screening Center Integrative Ortholog Prediction Tool (DIOPT) ( Hu *et al*. 2011), which integrates orthologue predictions for human and eight model organisms obtained from a range of popular orthology delineation tools. For comparisons beyond the core model species, FlyBase displays orthology predictions for other *Drosophila* species as well as for other selected arthropods sourced from the OrthoDB catalogue of orthologues ( Zdobnov *et al*. 2021). Other publicly available orthology resources containing predictions across multiple drosophilids and hundreds to thousands of other species include eggNOG v5.0 ( Huerta-Cepas *et al*. 2019), OrthoInspector ( Nevers *et al*. 2019), Ensembl Genomes ( Yates *et al*. 2022), and the OMA Orthologous Matrix browser ( Altenhoff *et al*. 2021). Most other online orthology resources emphasise taxonomic breadth over depth of sampling within a given lineage, and therefore usually only *D. melanogaster* is represented.

To take advantage of the growing number of available genome assemblies for *Drosophila* species, and to address the lack of orthology resources supporting genus-spanning multi-species comparative analyses to study fruit fly gene and genome evolution, we developed DrosOMA - the *Drosophila* Orthologous Matrix browser. DrosOMA uses the OMA ( Altenhoff *et al*. 2021) methodology to delineate orthology and paralogy for 36 drosophilids and four outgroup dipterans with high quality genome assemblies and annotations. The results are browsable in a feature-rich web interface, with gene-, orthologous group-, and genome-centric pages, as well as protein domain architecture and local and global genomic synteny visualisations, extensive gene name and identifier cross-references, and available functional annotations. This demonstrates the deployability of the OMA browser framework for building taxon-targeted orthology databases, here at the genus level, and provides a tailored resource for the *Drosophila* research community.

## Methods

### Species selection and annotation sources

*Drosophila* species with high quality and complete assembled and annotated genomes were selected for inclusion in DrosOMA so as to sample broadly across the genus. Of more than 350 assemblies representing some 140 species at the United States National Center for Biotechnology Information (NCBI), genome annotations were available for 49 species ( Sayers *et al*. 2023). Protein-coding gene annotations for *D. melanogaster* were sourced from FlyBase ( Gramates *et al*. 2022). All of the source data are available publicly - the accession numbers and version numbers are all given in Table 1. Assessments of completeness performed using Benchmarking Universal Single-Copy Orthologues ( BUSCO) ( R.M. Waterhouse *et al*. 2018; Manni *et al*. 2021) v5.4.0 and sourced from the A ^3^Cat Arthropoda Assembly Assessment Catalogue ( Feron and Waterhouse 2022) were used to select only annotated assemblies with Diptera-level BUSCO completeness scores of more than 95%. Filtering to reduce sampling of closely related species resulted in a final set of 36 *Drosophila* species with high-quality annotated assemblies for orthology delineation, as well as four outgroup mosquito species ( Table 1).

**Table 1.** Summary information of protein-coding gene annotation data used for orthology delineation. Annotations were sourced from the NCBI, apart from *D. melanogaster* annotations which were sourced from FlyBase. Only one isoform per gene is used as input for OMA.

Species	Code	Assembly Accession	Annotation Version	Number of Genes	BUSCO Assembly C [S,D]F,M	BUSCO Annotation C [S,D]F,M
*Aedes aegypti*	AEDAE	GCF_002204515.2	101	14,626	96.8 [93.5, 3.3], 1.6, 1.6	99.3 [95.4, 3.9], 0.2, 0.5
*Anopheles albimanus*	ANOAL	GCF_013758885.1	100	11,565	96.7 [96.4, 0.3], 0.9, 2.4	99.2 [98.7, 0.5], 0.2, 0.6
*Anopheles coluzzii*	ANOCL	GCF_013141755.1	100	12,592	97.1 [96.7, 0.4], 0.8, 2.1	99.3 [98.7, 0.6], 0.2, 0.5
*Anopheles stephensi*	ANOST	GCF_016920705.1	100	12,692	97.2 [93.5, 3.7], 0.9, 1.9	99.4 [95.2, 4.2], 0.1, 0.5
*Drosophila albomicans*	DROAB	GCF_009650485.1	100	13,590	96.6 [96.1, 0.5], 0.3, 3.1	97.6 [96.7, 0.9], 0.0, 2.4
*Drosophila ananassae*	DROAN	GCF_003285975.2	101	14,128	99.1 [98.8, 0.3], 0.5, 0.4	99.8 [99.5, 0.3], 0.0, 0.2
*Drosophila arizonae*	DROAR	GCF_001654025.1	100	12,476	95.4 [95.1, 0.3], 1.1, 3.5	95.6 [95.2, 0.4], 1.4, 3.0
*Drosophila biarmipes*	DROBM	GCF_000233415.1	101	14,230	98.9 [98.6, 0.3], 0.5, 0.6	99.8 [99.6, 0.2], 0.1, 0.1
*Drosophila bipectinata*	DROBP	GCF_000236285.1	101	14,981	98.7 [98.2, 0.5], 0.6, 0.7	99.3 [98.8, 0.5], 0.4, 0.3
*Drosophila busckii*	DROBS	GCF_011750605.1	101	12,712	97.4 [96.7, 0.7], 0.6, 2.0	98.0 [97.3, 0.7], 0.4, 1.6
*Drosophila elegans*	DROEL	GCF_000224195.1	101	15,407	98.8 [98.6, 0.2], 0.5, 0.7	99.7 [99.5, 0.2], 0.1, 0.2
*Drosophila erecta*	DROER	GCF_003286155.1	101	13,718	99.2 [98.8, 0.4], 0.3, 0.5	99.9 [99.5, 0.4], 0.0, 0.1
*Drosophila eugracilis*	DROEU	GCF_000236325.1	101	15,375	99.0 [98.7, 0.3], 0.4, 0.6	99.8 [99.6, 0.2], 0.1, 0.1
*Drosophila ficusphila*	DROFC	GCF_000220665.1	101	15,062	99.1 [98.6, 0.5], 0.6, 0.3	99.8 [99.3, 0.5], 0.1, 0.1
*Drosophila grimshawi*	DROGR	GCF_000005155.2	102	13,754	99.0 [96.7, 2.3], 0.4, 0.6	99.7 [97.3, 2.4], 0.2, 0.1
*Drosophila guanche*	DROGU	GCF_900245975.1	100	13,307	98.9 [98.4, 0.5], 0.6, 0.5	99.6 [99.2, 0.4], 0.1, 0.3
*Drosophila hydei*	DROHY	GCF_003285905.1	101	13,282	98.9 [97.0, 1.9], 0.5, 0.6	99.8 [97.5, 2.3], 0.1, 0.1
*Drosophila innubila*	DROIU	GCF_004354385.1	100	13,595	99.0 [98.6, 0.4], 0.4, 0.6	99.7 [99.1, 0.6], 0.1, 0.2
*Drosophila kikkawai*	DROKI	GCF_000224215.1	101	15,096	98.3 [97.4, 0.9], 0.7, 1.0	99.6 [98.8, 0.8], 0.2, 0.2
*Drosophila mauritiana*	DROMA	GCF_004382145.1	100	14,112	99.1 [98.8, 0.3], 0.5, 0.4	100.0 [99.5, 0.5], 0.0, 0.0
*Drosophila melanogaster*	DROME	GCF_000001215.4	6.32	13,968	98.7 [98.5, 0.2], 0.5, 0.8	100.0 [99.7, 0.3], 0.0, 0.0
*Drosophila miranda*	DROMI	GCF_003369915.1	102	19,112	98.9 [82.6, 16.3], 0.8, 0.3	99.7 [85.5, 14.2], 0.1, 0.2
*Drosophila mojavensis*	DROMO	GCF_000005175.2	101	13,329	98.8 [98.4, 0.4], 0.5, 0.7	99.5 [99.1, 0.4], 0.3, 0.2
*Drosophila navojoa*	DRONA	GCF_001654015.2	101	13,082	98.2 [97.9, 0.3], 0.9, 0.9	98.7 [98.3, 0.4], 0.6, 0.7
*Drosophila novamexicana*	DRONM	GCF_003285875.2	100	13,260	98.3 [97.5, 0.8], 0.4, 1.3	98.9 [98.1, 0.8], 0.1, 1.0
*Drosophila obscura*	DROOB	GCF_002217835.1	100	16,865	98.8 [94.4, 4.4], 0.6, 0.6	99.3 [94.6, 4.7], 0.2, 0.5
*Drosophila persimilis*	DROPE	GCF_003286085.1	101	14,397	98.8 [97.2, 1.6], 0.8, 0.4	99.7 [97.8, 1.9], 0.0, 0.3
*Drosophila pseudoobscura*	DROPS	GCF_009870125.1	104	14,343	98.7 [98.0, 0.7], 0.9, 0.4	99.7 [98.8, 0.9], 0.1, 0.2
*Drosophila rhopaloa*	DRORH	GCF_000236305.1	101	16,017	97.5 [96.4, 1.1], 1.4, 1.1	98.3 [97.0, 1.3], 1.0, 0.7
*Drosophila santomea*	DROSN	GCF_016746245.1	100	14,039	98.6 [98.4, 0.2], 0.4, 1.0	99.9 [99.6, 0.3], 0.0, 0.1
*Drosophila sechellia*	DROSE	GCF_004382195.1	101	14,182	99.2 [98.7, 0.5], 0.4, 0.4	99.9 [99.3, 0.6], 0.0, 0.1
*Drosophila serrata*	DROSR	GCF_002093755.1	100	14,775	97.2 [95.3, 1.9], 1.8, 1.0	99.9 [97.5, 2.4], 0.0, 0.1
*Drosophila simulans*	DROSI	GCF_016746395.1	102	14,143	99.0 [98.8, 0.2], 0.4, 0.6	99.9 [99.4, 0.5], 0.0, 0.1
*Drosophila subobscura*	DROSU	GCF_008121235.1	100	13,440	98.7 [98.2, 0.5], 0.7, 0.6	99.7 [99.1, 0.6], 0.1, 0.2
*Drosophila subpulchrella*	DROSH	GCF_014743375.2	100	15,028	98.9 [98.2, 0.7], 0.6, 0.5	99.9 [99.0, 0.9], 0.0, 0.1
*Drosophila suzukii*	DROSZ	GCF_013340165.1	102	15,567	97.3 [94.5, 2.8], 1.5, 1.2	99.8 [96.6, 3.2], 0.1, 0.1
*Drosophila takahashii*	DROTK	GCF_000224235.1	101	15,410	98.8 [98.1, 0.7], 0.5, 0.7	99.7 [99.0, 0.7], 0.2, 0.1
*Drosophila virilis*	DROVI	GCF_003285735.1	103	13,685	99.1 [97.3, 1.8], 0.5, 0.4	99.8 [97.7, 2.1], 0.1, 0.1
*Drosophila willistoni*	DROWI	GCF_000005925.1	101	13,769	98.8 [97.9, 0.9], 0.3, 0.9	99.8 [98.9, 0.9], 0.0, 0.2
*Drosophila yakuba*	DROYA	GCF_016746365.1	101	14,085	99.0 [98.8, 0.2], 0.4, 0.6	99.7 [99.5, 0.2], 0.1, 0.2

### Orthology delineation using OMA

All annotated protein-coding genes from the 40 selected species were used as input for delineating orthologous groups for DrosOMA. Briefly, orthology delineation using the OMA Standalone inference algorithm consists of three main stages ( Altenhoff *et al*. 2019, 2021). Firstly, all-against-all Smith-Waterman sequence alignments are computed using the SWPS3 vectorized implementation of the Smith-Waterman local alignment algorithm and significant matches are retained to define homologous proteins (i.e. sequences with a common ancestry). Before inferring orthology, one representative protein per gene is selected. OMA Standalone uses all isoforms for the first all-against-all alignment stage and selects as the reference protein the isoform with the best matches across all species (this can be considered as the most evolutionarily conserved isoform). Secondly, mutually closest homologues between species pairs are identified based on evolutionary distances to infer orthologous pairs (i.e. homologues related through speciation), while accounting for distance inference uncertainties and for potential differential gene losses. Finally, all identified orthologous pairs are clustered using two different approaches to produce catalogues of OMA Groups and Hierarchical Orthologous Groups (HOGs) ( Zahn-Zabal *et al*. 2020). HOGs are defined as sets of genes that descended from a single ancestral gene at a given taxonomic range. These sets correspond to the idea of subfamilies for a given taxonomic range and can contain more than one gene from a species, i.e. inparalogues. OMA Groups on the other hand are sets of orthologues where each gene is orthologous to one another. The history of such sets should correspond to the species phylogeny and hence they are especially useful as markers to reconstruct the species phylogeny. For this dataset the production pipeline of OMA was employed, but the same clustering can also be performed using OMA standalone. In order to build the browsable DrosOMA instance, the OMA orthologues were converted using the oma2hdf command from the pyoma python package into an HDF5 database. CATH domain annotations ( Sillitoe *et al*. 2021) were computed using the cath-tools v0.16.10 package and with the provided hmm models from CATH release 4.2. Protein cross-references were added by matching the sequences against the full UniProtKB and RefSeq databases, requiring exact matches.

### Web server virtual machine configuration and setup

The OMA browser instance for DrosOMA was set up and is hosted on a virtual machine using docker containers. The virtual machine requires relatively modest resources, i.e. 2 CPUs clocked at 2.25 GHz each, 8 GB RAM and 25 GB storage. The docker images for the OMA Browser were created from the pyomabrowser repository ( https://github.com/DessimozLab/pyomabrowser) following the steps described in https://zoo.cs.ucl.ac.uk/doc/pyomabrowser/setup.html. Before building the docker images, the following aspects of the OMA Browser web interface were adjusted in order to make it a *Drosophila*-specific instance: We removed all the instances of non-drosophila proteins in the search examples by adjusting the Django templates in oma/templates, oma/test/ and oma_rest/. Similarly, we changed the OMA logo by replacing the corresponding file in/oma/static/image. These customisations are mostly cosmetic changes that will make the service more user friendly, and are not strictly needed for website functionality. Finally, paths, deployment type, and rabbitmq/celery credentials were adjusted and hosts were allowed in for_docker/env.

### Species phylogeny reconstruction

The species tree was computed using single-copy orthologues identified during the BUSCO completeness assessments of the genomes of the species selected for inclusion in DrosOMA. The protein sequences of BUSCO genes found in at least 38 of the 40 species were aligned using MUSCLE 3.8.1551 ( Edgar 2004) with default settings and subsequently trimmed to retain well-aligned regions using TrimAl ( Capella-Gutiérrez *et al*. 2009) with the “-strictplus” option. The 2,891 alignments were merged to build a 40-species concatenated superalignment (1,581,953 columns; 683,285 distinct patterns; 658,691 parsimony-informative; 180,333 singleton sites; 742,929 constant sites) used as input for phylogeny reconstruction using IQ-TREE 2.2.0-beta ( Nguyen *et al*. 2015) with 1,000 bootstrap samples (options: -msub nuclear -B 1000 -bnni). The molecular species phylogeny was time-calibrated by providing calibration dates for the Diptera root, Culicidae, Drosophilini, willistoni-melanogaster ancestor, and navojoa-albomicans ancestor, from the TimeTree database ( Kumar *et al*. 2022) to the functions makeChronosCalib() and chronos(), from the ape R package ( Paradis and Schliep 2019), and plotted using the ggtree R package ( Yu 2023).

### Implementation

The DrosOMA Drosophila Orthologous Matrix browser implements for users a feature-rich web interface to explore the results of orthology inference amongst complete genomes. The service is implemented with the django framework, a high-level Python web framework that encourages rapid development and clean, pragmatic design.

### Operation

The DrosOMA Drosophila Orthologous Matrix browser operates on standard up-to-date web browsers including Google Chrome, Mozilla Firefox, and Apple Safari. The operational setup of an OMA browser instance such as DrosOMA requires a host that runs docker containers orchestrated with docker compose.

## Results

### Orthologous groups delineated across 36 Drosophila species

Applying OMA orthology delineation to the protein-coding genes from 36 drosophilids and four outgroup mosquito species (see Methods) resulted in the clustering of 93.5% of proteins in OMA Groups and 95.6% in Hierarchical Orthologous Groups (HOGs), with almost 25,000 HOGs at the last common ancestor of all DrosOMA species ( Table 2). The OMA Groups are cliques of orthologues based on the orthology graph, meaning that all the components (proteins) of an OMA Group are connected to each other through pairwise orthologous relationships. Although all members of the OMA Groups are orthologous to all other members of the same group, OMA group members are not necessarily 1-to-1 orthologues. The OMA HOGs comprise sets of proteins encoded by genes descended from a common ancestral gene in the last common ancestor of a set of species (i.e. at a specific taxonomic level in the species phylogeny). The “hierarchical” nature of HOGs is due to their being defined with respect to specific clades within the species tree, so HOGs are nested subfamilies with groups delineated for younger radiations being encompassed within larger HOGs defined at older nodes. DrosOMA contains HOGs delineated at the root, three mosquito nodes, and 13 drosophilid nodes including Sophophora, the melanogaster group, and the melanogaster subgroup.

**Table 2.** Summary statistics of DrosOMA orthology delineation results.

Feature	Count
Number of species	40
Total number of proteins	568,796
Number of OMA Groups	962,065
Number of proteins in OMA Groups	531,644 (93.5%)
Number of root-level HOGs	24,896
Number of proteins in HOGs	544,034 (95.6%)
Number of universal single-copy orthologues	2,428
Number of proteins mapped to UniProt	309,657 (54.4%)
Number of proteins mapped to Gene Ontology terms	350,568 (61.6%)

**Figure 1.**
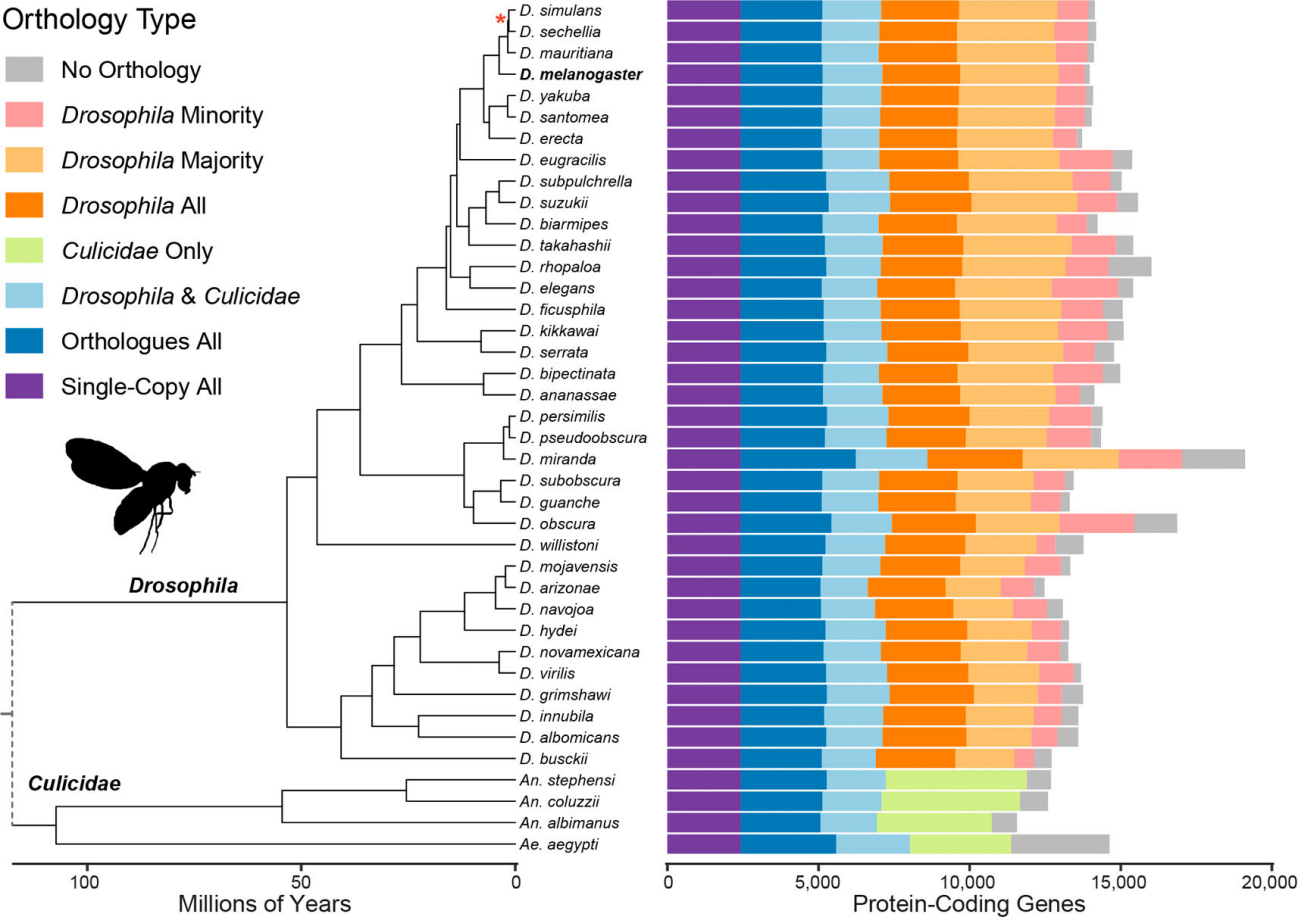
Species phylogeny and orthology classifications across 36 *Drosophila* and four outgroup species. The time-calibrated species phylogeny (left) shows the estimated evolutionary relationships amongst the set of 40 species spanning approximately 60 million years since the last common ancestor of the *Drosophila* genus. The dashed line indicates the *Drosophila* and *Culicidae* last common ancestor but for visualisation is not placed according to the timescale. The barchart (right) shows counts of genes per species categorised according to their orthology type based on root-level hierarchical orthologous groups (HOGs). Analysis of the root-level HOGs shows counts of proteins per species belonging to universal single-copy HOGs (Single-copy All), universal but variable-copy-number HOGs (Orthologues All), non-universal HOGs with outgroup species orthologues ( *Drosophila* & *Culicidae*), mosquito-only orthologues ( *Culicidae* Only), as well as drosophilid-specific HOGs with orthologues from all ( *Drosophila* All), the majority ( *Drosophila* Majority), or the minority ( *Drosophila* Minority) of the 36 Drosophila species. This leaves an average of 527 ±392 proteins per drosophilid species with no identifiable orthologues, i.e. annotated protein-coding genes that, given the set of species under consideration, appear to be species-specific with no traceable common ancestry. Branch lengths are shown in millions of years; all nodes received 100% bootstrap support except * with 95%; *D. Drosophila*; *An. Anopheles*; *Ae. Aedes*; Minority <18 drosophilids; Majority ≥18 drosophilids.

The fully-resolved time-calibrated species phylogeny (see Methods) defines the relationships amongst the 36 *Drosophila* species and the outgroup mosquitoes over approximately 260 million years of evolution ( Figure 1). Analysis of the root-level HOGs shows counts of proteins per species belonging to universal single-copy HOGs (9.8% of HOGs; 17.1% of proteins), universal but variable-copy-number HOGs (19.6% of proteins), non-universal HOGs with outgroup species orthologues (13.7% of proteins), as well as drosophilid-specific HOGs with orthologues from all (16.8% of proteins), the majority (17.9% of proteins), or the minority (7.7% of proteins) of the 36 *Drosophila* species. This leaves an average of 527±392 proteins per drosophilid species with no identifiable orthologues, i.e. annotated protein-coding genes that, given the set of species under consideration, appear to be species-specific with no traceable common ancestry.

### Orthology data exploration using the DrosOMA browser

**Figure 2.**
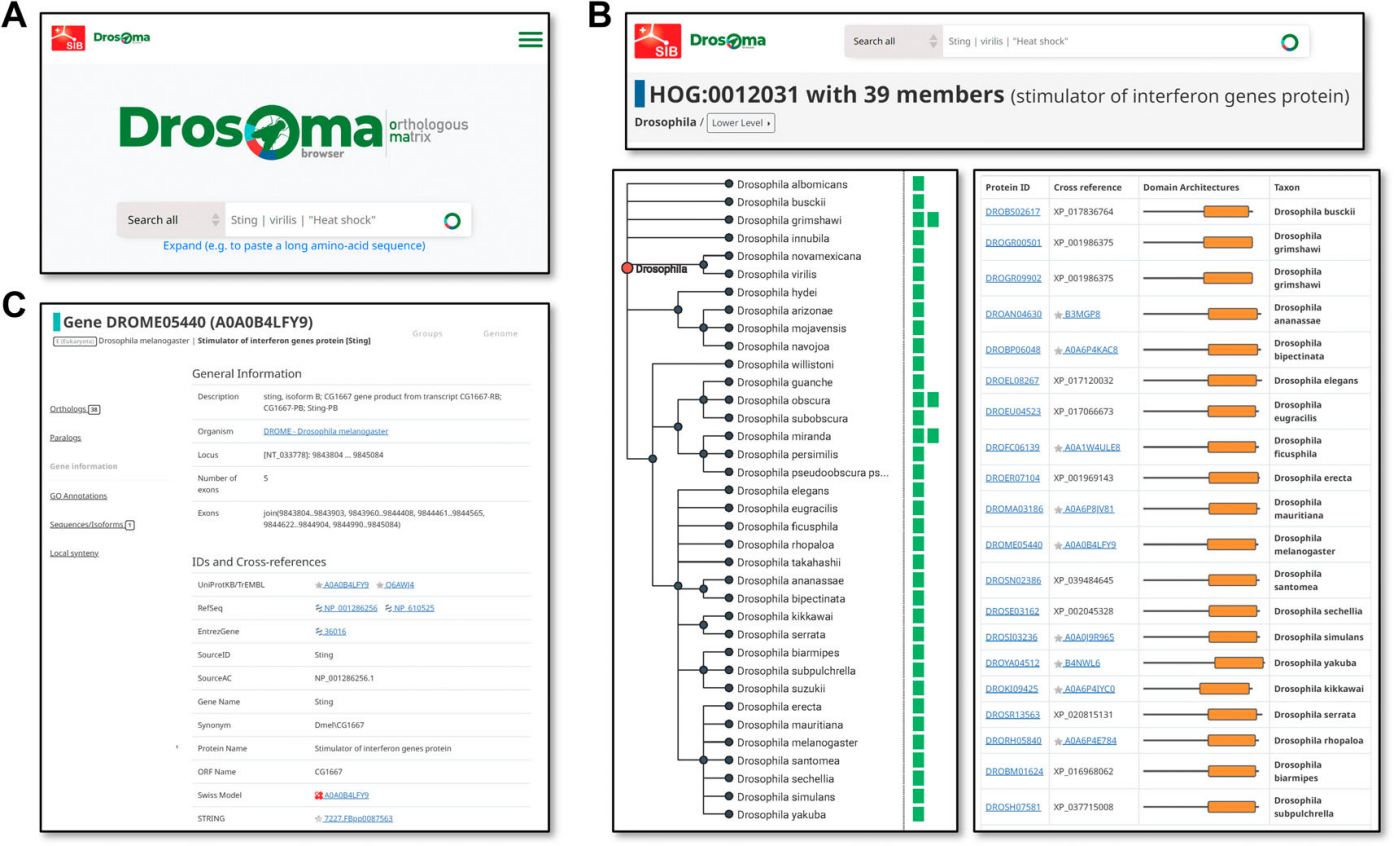
Example orthologous group and gene information views available from the DrosOMA browser. (A) The simple search entry point for DrosOMA allows for text searches with gene names, descriptors, or identifiers, as well as with protein sequences. (B) Visualising information for Hierarchical Orthologous Groups (HOGs) can be guided by the species phylogeny (left) showing counts of orthologues per species, or as a table (right) with protein identifiers and cartoons showing domain architectures. (C) The gene view page displays available information for genes of interest and their mappings to external databases.

As DrosOMA uses the same database and interface design and architecture as the OMA browser ( Altenhoff *et al*. 2021), an extensive array of data querying and visualisation options are available to the user. Searches may be performed using gene or protein names, descriptors, or identifiers, or protein sequences, and extensive cross-referencing to public databases allows for searches using identifiers from resources such as UniProt ( The UniProt Consortium *et al*. 2023), RefSeq ( O’Leary *et al*. 2016), EntrezGene ( Sayers *et al*. 2023), Swiss Model ( A. Waterhouse *et al*. 2018), STRING ( Szklarczyk *et al*. 2023), and Bgee ( Bastian *et al*. 2021), in addition to the source FlyBase and NCBI identifiers and annotations ( Figure 2A). Search result visualisations are focused on the three main data types, i.e. with views for genomes, groups ( Figure 2B), or genes ( Figure 2C). Genome-view pages summarise available information per species, e.g. a list of all their genes and of their most closely related species, as well as tools for building pairwise global synteny visualisations. Group-view pages display information about OMA Groups or HOGs, showing filterable lists of member genes with their associated cross-referenced identifiers and cartoon views of protein domain architectures, as well as visualisations of HOG members guided by the species phylogeny. Gene-view pages display information associated with a gene and its protein products, including sequences (protein and cDNA), cross references to other public databases, and available functional annotations in the form of Gene Ontology terms ( The Gene Ontology Consortium *et al*. 2021).

**Figure 3.**
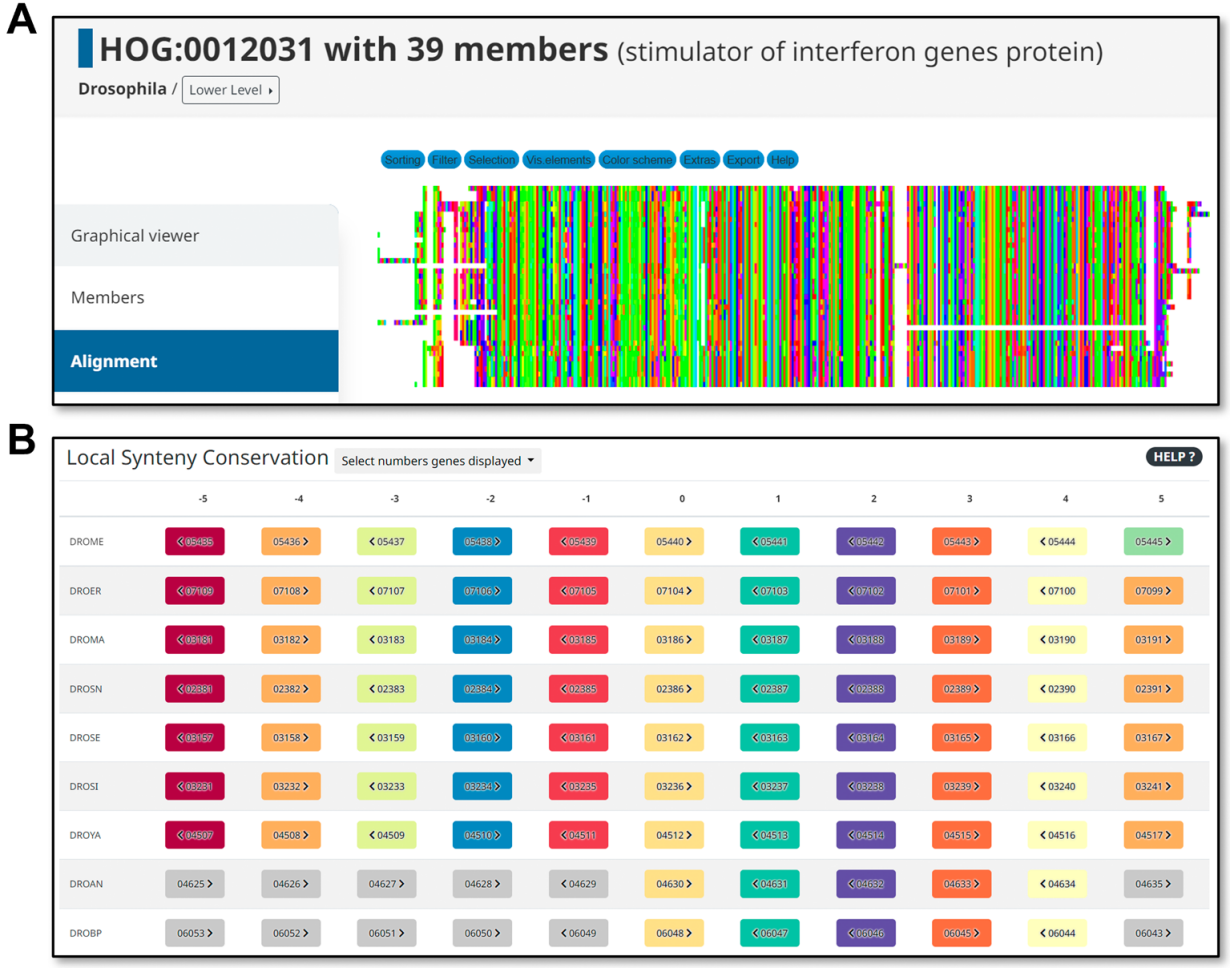
Example additional analysis views available from the DrosOMA browser. (A) Multiple sequence alignments of proteins from hierarchical orthologous groups (HOGs) or OMA Groups can be generated, visualised, explored, and downloaded using the DrosOMA Browser. (B) Local gene synteny conservation can be visualised to explore how orthologues have maintained or shuffled their local arrangements in the genomes of each considered species.

Other useful search, visualisation, and download features are described in the DrosOMA “Explore”, “Tools”, “Download”, and “Help” pages, with several examples and explanations for the general use of the OMA browser elaborated in a dedicated primer ( Zahn-Zabal *et al*. 2020). Examples of these extended features include sequence alignment tools ( Figure 3A) and local synteny visualisations ( Figure 3B). For both OMA Groups and HOGs, the browser can generate multiple sequence alignments of the member proteins that can further be sorted, filtered, edited, and exported by users, for example, to use as inputs for building gene trees for orthologous groups of interest. Synteny, or how orthologues have maintained or shuffled their genomic arrangements throughout evolution, can be visualised at a local level (e.g. from a context of 9 to 19 orthologues) or at global level (along entire chromosomes for pairs of species), both based on comparing the relative genomic positions of orthologues across the species under consideration.

## Conclusions

The rapidly growing number of species with sequenced and annotated genomes mean that publicly accessible resources offering results from large-scale comparative analyses such as orthology delineation often prioritise taxonomic breadth over depth when selecting which species to include. This means that despite increasingly comprehensive species sampling within some taxonomic groups, the available genomic data can remain under-exploited as only representative species are included in most taxonomically broad resources. The DrosOMA browser provides a resource aimed at the *Drosophila* research community that exploits the available high-quality genome annotation data across the genus. The successful deployment of DrosOMA illustrates the feasibility and utility of the OMA browser framework to be applied to other taxonomic groups with rapidly growing numbers of species with genomic data. Future studies taking advantage of increased taxonomic depth of sampling within a given genus, such as previous genus-wide investigations of *Anopheles* mosquitoes ( Neafsey *et al*. 2015) or *Bombus* bumblebees ( Sun *et al*. 2021), could therefore benefit from applying the framework to not only obtain orthology data, but to simultaneously build and deploy an interactive browser to further support their research. Yet-to-be annotated genome assemblies are publicly available for almost 100 more drosophilids, and data generation for additional species is ongoing. As more high-quality annotations for high-quality genomes become publicly available, future DrosOMA releases are set to further deepen taxonomic representation within the genus containing the arguably best studied representative of all animals.

## Data Availability

The underlying data is available from the DrosOMA Browser ( https://drosoma.dcsr.unil.ch/). All sequence data used to build the DrosOMA browser database were originally sourced from a public repository, the United States National Center for Biotechnology Information (NCBI). The sources for which have been compiled and are provided on an online repository below: Figshare: Table S1: Data Sources for DrosOMA, the Drosophila Orthologous Matrix browser. https://doi.org/10.6084/m9.figshare.23622507.v1 ( Thiébaut *et al.*, 2023). This project contains the following underlying data:
-
DrosOMA_Data_TableS1.xlsx (Data Sources for DrosOMA, the Drosophila Orthologous Matrix browser). Data are available under the terms of the Creative Commons Attribution 4 International (CC BY 4.0) license. The underlying sequences and annotations from the NCBI may be subject to third-party constraints (some submitters of the original data, or the country of origin of such data, may claim patent, copyright, or other intellectual property rights in all or a portion of the data). Users of the data are solely responsible for establishing the nature of, and complying with, any such intellectual property restrictions, as the authors of this article have done. The completeness assessments used to select high-quality public data were sourced from the A ^3^Cat Arthropoda Assembly Assessment Catalogue.

## Software availability

DrosOMA browser available at: https://drosoma.dcsr.unil.ch/.

Source code available from: https://github.com/DessimozLab/OmaStandalone (orthology inference) and https://github.com/DessimozLab/pyomabrowser (Django based webserver)

Archived source code at time of publication: https://zenodo.org/record/8028421

Licence: Mozilla Public License 2.0
