# Supplementary material for: DrosOMA: the Drosophila Orthologous Matrix browser
Source: F1000Res. 2024 Jan 16;12:936. Originally published 2023 Aug 7. [Version 2] doi: 10.12688/f1000research.135250.2 (PMC10905159; doi:10.12688/f1000research.135250.2)
Supplement: Peer review history

# Reviewer report: Reviewer response for version 2

**DOI:** 10.5256/f1000research.161318.r248494

**Published:** 2024-03-04

**Authors:** Elise Parey

**Affiliations:** 1 University College London (Ringgold ID: 4919), London, England, UK

In this study, Antonin Thiébaut and co-authors introduce DrosOMA - the Drosophila Orthologous Matrix browser, a unique resource for comparative genomics across *Drosophila* species. DrosOMA fills an important gap in the tools currently available to the *Drosophila* research community, as the available resources are heavily centered around the *Drosophila melanogaster* model. The resource is also timely given the increasing availability of high-quality genomic resources for species in this group. I found the manuscript well-written, clear and concise, with the DrosOMA browser being efficient, well-documented and intuitive to use. I only have a few minor comments and questions:

1. It would be interesting to indicate which of the 36 *Drosophila* and four outgroup genomes are chromosome-scale assemblies, perhaps in Table 1? I think this would be relevant to interpret the synteny visualizations that are available on the browser
2. The authors reconstructed a time-calibrated phylogeny for the 40 considered species. I was wondering whether this result was new or confirming previous phylogenetic studies in this group?
3. This is more a question/suggestion regarding future developments, but I was wondering if, for the local synteny view, it would be possible to make more gene information appear on mouse hover, for instance (putative) gene or protein names?

Are the conclusions about the tool and its performance adequately supported by the findings presented in the article?

Yes

Is the rationale for developing the new software tool clearly explained?

Yes

Is the description of the software tool technically sound?

Yes

Are sufficient details of the code, methods and analysis (if applicable) provided to allow replication of the software development and its use by others?

Yes

Is sufficient information provided to allow interpretation of the expected output datasets and any results generated using the tool?

Yes

Reviewer Expertise:

Phylogenomics, Animal Comparative Genomics, Synteny

I confirm that I have read this submission and believe that I have an appropriate level of expertise to confirm that it is of an acceptable scientific standard.

# Reviewer report: Reviewer response for version 2

**DOI:** 10.5256/f1000research.161318.r243760

**Published:** 2024-02-28

**Authors:** Jung-Wan Mok

**Affiliations:** 1 Texas Children's Hospital, Texas Medical Center (Ringgold ID: 3973), Houston, Texas, USA; 2 Baylor College of Medicine, Houston, Texas, USA

The authors of the article introduced the DrosOMA browser, which enables users to access genome/protein data of quality. As the authors claimed, previous browsers, including Flybase (which is fly-specific) or the Orthologous Matrix browser (OMA), already provided brief access to some Drosophila species, but it was not comprehensive nor user-friendly except for the most famous species, 'Drosophila melanogaster'.

 As a Drosophila geneticist, I find the browser to be beneficial to many researchers. One wonderful feature of the browser is that it provides multi-species protein alignment, as shown in Figure 3. This enables users to identify protein loci that are prone to evolutionary pressure. Additionally, this feature could be beneficial to researchers using Drosophila as a human disease model, as they can further evaluate the VUS/allele of their interest.

 The only minor suggestion I have is about the 'consensus sequence' function. There is no explanation about how to add the consensus sequence manually, so I tried multiple times to figure this out. Also, when the user adds a 'consensus sequence' to the matrix, the added sequence disappears when the user clicks on any region of the figure. Furthermore, there is a typo – when the consensus sequence is added, it becomes 'consenus sequence' instead of 'consensus'.

 I do not have any other comments on the manuscript.

Are the conclusions about the tool and its performance adequately supported by the findings presented in the article?

Yes

Is the rationale for developing the new software tool clearly explained?

Yes

Is the description of the software tool technically sound?

Yes

Are sufficient details of the code, methods and analysis (if applicable) provided to allow replication of the software development and its use by others?

Yes

Is sufficient information provided to allow interpretation of the expected output datasets and any results generated using the tool?

Partly

Reviewer Expertise:

Drosophila Genetics / Developmental Biology / Neuroscience / Disease Model / Tool generation

I confirm that I have read this submission and believe that I have an appropriate level of expertise to confirm that it is of an acceptable scientific standard.

# Reviewer report: Reviewer response for version 2

**DOI:** 10.5256/f1000research.161318.r238295

**Published:** 2024-02-23

**Authors:** Berend Snel

**Affiliations:** 1 Utrecht University, Utrecht, The Netherlands

The authors have answered, implemented and evaluated all comments with great care.

Are the conclusions about the tool and its performance adequately supported by the findings presented in the article?

Partly

Is the rationale for developing the new software tool clearly explained?

Yes

Is the description of the software tool technically sound?

Yes

Are sufficient details of the code, methods and analysis (if applicable) provided to allow replication of the software development and its use by others?

Yes

Is sufficient information provided to allow interpretation of the expected output datasets and any results generated using the tool?

Partly

Reviewer Expertise:

Comparative genomics. Bioinformatics. Orthology. Evolution of protein complexes.

I confirm that I have read this submission and believe that I have an appropriate level of expertise to confirm that it is of an acceptable scientific standard.

# Reviewer report: Reviewer response for version 1

**DOI:** 10.5256/f1000research.148360.r222942

**Published:** 2023-12-20

**Authors:** Daofeng Li

**Affiliations:** 1 Department of Genetics, Washington University in St Louis, St. Louis, Missouri, USA

The authors described a well-developed web portal to allow users query high-quality gene/protein annotations across drosophila genus (and 4 others). The data for ontology annotations used on the website were generated by the published method OMA. The website is fast and user friendly, the manuscript and figures are well written/designed. This could be a valuable resource for the fruit fly research community.  Meanwhile, docker container is provided to enable users generate annotations and web interface from their interested species.

 I only have some minor suggestions:

 1) In the query box of the DrosOMA landing page, I hope the authors can implement an example data button to add example query input the search box, especially when user changes the item from the dropdown menu, the suggested example query item should update.

 2) The documentation for the DrosOMA website is minimal, if the users can expand the documentation for the website could be great, like how to use the website, how to explain the results, how to download a plot etc.

 3) I am wondering if the OMA method could work on other types of gene except protein coding genes, such as rRNA gene, pseudo genes etc.

 4) Could the author explain more about how to update the annotation data when trying to add/remove/update any source genomes/annotations.

Are the conclusions about the tool and its performance adequately supported by the findings presented in the article?

Yes

Is the rationale for developing the new software tool clearly explained?

Yes

Is the description of the software tool technically sound?

Yes

Are sufficient details of the code, methods and analysis (if applicable) provided to allow replication of the software development and its use by others?

Yes

Is sufficient information provided to allow interpretation of the expected output datasets and any results generated using the tool?

Yes

Reviewer Expertise:

Data visualization, epigenomics, Genome Browser

I confirm that I have read this submission and believe that I have an appropriate level of expertise to confirm that it is of an acceptable scientific standard.

# Response

**Published:** 2024-01-11

**Authors:** Robert Waterhouse

**Affiliations:** University of Geneva, Switzerland

The authors described a well-developed web portal to allow users query high-quality gene/protein annotations across drosophila genus (and 4 others). The data for ontology annotations used on the website were generated by the published method OMA. The website is fast and user friendly, the manuscript and figures are well written/designed. This could be a valuable resource for the fruit fly research community.  Meanwhile, docker container is provided to enable users generate annotations and web interface from their interested species.

**=> We thank the reviewer for their appreciative summary comments.**

 I only have some minor suggestions:

 1) In the query box of the DrosOMA landing page, I hope the authors can implement an example data button to add example query input the search box, especially when user changes the item from the dropdown menu, the suggested example query item should update.

**=> While a few generic examples are provided in the search box and additional examples below, it could indeed be useful to add updatable terms that match the types of query available from the dropdown menu. As this would be specific to each instance of an OMA browser (depending on species, versions etc.) adding this would make more curation work necessary for anyone wishing to build an OMA browser for themselves, so we aim to keep it simple and minimise the customisation burden.**

 2) The documentation for the DrosOMA website is minimal, if the users can expand the documentation for the website could be great, like how to use the website, how to explain the results, how to download a plot etc.

**=> The menus on the top right of the pages (three horizontal bars on small screens, full drop-down menus on larger screens) provide quick and easy access to the documentation, this includes pages dedicated to how to explore the database, the tools connected to using DrosOMA and OMA in general, a download page showing how to obtain the data, as well as the main help pages - Introduction, Orthology basics; Type of homologs; Access the OMA data; Catalog of tools; FAQ; Q&A on Biostars; the Glossary; the link to the OMA Academy pages; and finally, the About page. At the bottom of the home page there is further help information, e.g. in the box titled “First time here?”, as well as the “OMA tools” and “Download options” information boxes. Several specific pages also provide more targeted help for the user, e.g. the alignment viewer page provides a Help button to access the user manual, the local synteny viewer provides a Help button showing a graphic explaining how the viewer works, etc. In addition, in the manuscript we also point to helpful information for users elaborated in a dedicated primer (Zahn-Zabal et al. 2020). To us this seems already very comprehensive, of course we can and will continue to elaborate these help pages in the context of updates to OMA and DrosOMA, especially to describe new features.**

 3) I am wondering if the OMA method could work on other types of gene except protein coding genes, such as rRNA gene, pseudo genes etc.

**=> In theory, if equivalent pairwise distances could be computed then the rest of the OMA algorithm could be applied to other types of genes, but the OMA method has not been applied to non-protein-coding genes and is not designed to process them.**

 4) Could the author explain more about how to update the annotation data when trying to add/remove/update any source genomes/annotations.

**=> For a comprehensive update, all pre-processing steps need to be re-computed, this is especially important because of the number of links to external sources provided by DrosOMA because these databases also update their information. So future releases will include more Drosophila species but the processing and orthology delineation needs to be completely rerun to ensure internal and external consistency and robustness.**

# Reviewer report: Reviewer response for version 1

**DOI:** 10.5256/f1000research.148360.r219663

**Published:** 2023-11-24

**Authors:** Berend Snel

**Affiliations:** 1 Utrecht University, Utrecht, The Netherlands

The article describes a novel browsable database for the OMA output specific for the Drosophila genus. The methods, including the more technical computational details and genomic considerations of completeness are very clear. The different features of the dataset that are available and how to browse them are also very clear.

 I have only a few points to discuss.

 First I am a bioinformatician myself and not a “drosophila” biologist. I am not sure if the current manuscript sufficiently explains to such an audience what the database and its interface exactly offer and what inferences you can use it for. Maybe collaboration with such an expert can be sought to see if they indeed see the data set as something for them.

 Second and this is quite big but not essential, we are currently basically redoing all of our normal sequence searches with AF2 structural searches. Especially for the inverse cases of “lineage specific genes” or “lineage specific gene loss” it turns out that a lot more homology and thereby also orthology remains to be discovered. I wonder if this is something to consider for the future (but I guess  that is an OMA issue and not a drosOMA issue).

 Finally given also the above considerations, panel B of figure 3 shows synteny and the final gene DROME05445 stands out in its column i.e. a green gene in an orange column. So I checked this situation in drosOMA which was indeed eminently browsable, and could open both that gene and its syntenic (but according to drosOMA non orthologous) gene in uniprot (which the the tools lets me do very easily), and the AF2 predictions are identical leading me to expect that both genes are potentially orthologous. In any case I am always worried about false negatives and the visualization via synteny might highlight potential false negatives.

Are the conclusions about the tool and its performance adequately supported by the findings presented in the article?

Partly

Is the rationale for developing the new software tool clearly explained?

Yes

Is the description of the software tool technically sound?

Yes

Are sufficient details of the code, methods and analysis (if applicable) provided to allow replication of the software development and its use by others?

Yes

Is sufficient information provided to allow interpretation of the expected output datasets and any results generated using the tool?

Partly

Reviewer Expertise:

Comparative genomics. Bioinformatics. Orthology. Evolution of protein complexes.

I confirm that I have read this submission and believe that I have an appropriate level of expertise to confirm that it is of an acceptable scientific standard, however I have significant reservations, as outlined above.

# Response

**Published:** 2024-01-11

**Authors:** Robert Waterhouse

**Affiliations:** University of Geneva, Switzerland

The article describes a novel browsable database for the OMA output specific for the Drosophila genus. The methods, including the more technical computational details and genomic considerations of completeness are very clear. The different features of the dataset that are available and how to browse them are also very clear.

**=> We thank the reviewer for the positive comments and for particularly recognising the clarity of the methods and features descriptions.**

 I have only a few points to discuss.

 First I am a bioinformatician myself and not a “drosophila” biologist. I am not sure if the current manuscript sufficiently explains to such an audience what the database and its interface exactly offer and what inferences you can use it for. Maybe collaboration with such an expert can be sought to see if they indeed see the data set as something for them.

**=> We thank the reviewer for bringing up this point about better explaining to potential users what they might use the resources for. After describing the main features we had only briefly mentioned the use of the data in large-scale genus-wide evolutionary studies, giving the Anopheles mosquitoes and Bombus bumblebees as examples. In the data exploration section we now added a few more specific examples to help explain the DrosOMA browser offerings in more concrete terms.**

 Second and this is quite big but not essential, we are currently basically redoing all of our normal sequence searches with AF2 structural searches. Especially for the inverse cases of “lineage specific genes” or “lineage specific gene loss” it turns out that a lot more homology and thereby also orthology remains to be discovered. I wonder if this is something to consider for the future (but I guess  that is an OMA issue and not a drosOMA issue).

**=> We agree that this is a very interesting topic and very relevant for inferences made about gene evolutionary histories that could be missing key information when real homology fails to be detected. Ongoing OMA development continues to address these and other issues, which are faced by all orthology delineation methods.**

 Finally given also the above considerations, panel B of figure 3 shows synteny and the final gene DROME05445 stands out in its column i.e. a green gene in an orange column. So I checked this situation in drosOMA which was indeed eminently browsable, and could open both that gene and its syntenic (but according to drosOMA non orthologous) gene in uniprot (which the the tools lets me do very easily), and the AF2 predictions are identical leading me to expect that both genes are potentially orthologous. In any case I am always worried about false negatives and the visualization via synteny might highlight potential false negatives.

**=> We thank the reviewer for taking the time to carry out this investigation and we are pleased that the browsing experience was easy and useful. We investigated this example and agree with the reviewer that these genes are both syntenic and orthologous. Investigating the rendering of the synteny visualisation revealed that the colour palette we had used did not provide enough colours for proper rendering, we have updated the palette and visualisation is now correct. We updated Figure 3 accordingly with a new screenshot of the local synteny visualisation.**
